# Fractional CO_2_ Laser Treatment Is Safe and Effective for the Management of Genitourinary Syndrome of Menopause in Korean Women

**DOI:** 10.3390/jcm12113679

**Published:** 2023-05-26

**Authors:** Youn-Jee Chung, Suhyun Shim, Sejin Kim, Jimin Cha, Jae-Yen Song, Min Jeong Kim, Mee-Ran Kim

**Affiliations:** 1 Department of Obstetrics and Gynecology, Seoul St. Mary’s Hospital, College of Medicine, The Catholic University of Korea, Seoul 06591, Republic of Korea; porshe80@catholic.ac.kr (Y.-J.C.); llhuyull@naver.com (S.S.); ksally37@gmail.com (S.K.); jmcatholic12@gmail.com (J.C.); jaeyen77@catholic.ac.kr (J.-Y.S.); 2 Department of Obstetrics and Gynecology, Bucheon St. Mary’s Hospital, College of Medicine, The Catholic University of Korea, Bucheon 14662, Republic of Korea; poouh74@catholic.ac.kr

**Keywords:** CO_2_ laser, menopause, genitourinary syndrome of menopause, vaginal dryness, vulvovaginal atrophy

## Abstract

This study evaluates the efficacy and safety of fractional CO_2_ lasers for treating genitourinary syndrome of menopause (GSM) in Korean women. The patients received three laser applications at an interval of 4 weeks each. The severity of GSM symptoms was assessed using a visual analog scale (VAS) at baseline and at every visit. The objective scale was measured using the vaginal health index score (VHIS) and Vaginal Maturation Index (VMI) after completion of the laser procedure. During each procedure, the patients’ pain in the VAS score was recorded. In the last visit, patients evaluated their satisfaction with the laser therapy using a 5-point Likert scale. Thirty women completed all the study protocols. After two sessions of laser therapy, some GSM symptoms (vaginal dryness and urgency) and VHIS improved significantly. After completion of the treatment, all GSM symptoms improved (*p* < 0.05), and the VHIS further increased significantly (VHIS at baseline, 8.86 ± 3.2 vs. V3, 16.83 ± 3.15, *p* < 0.001). The average satisfaction was 4.3. This study shows that fractional CO_2_ laser treatment is effective and safe for Korean women with GSM. Further studies are needed to confirm these results and assess the long-term effects of laser therapy.

## 1. Introduction

A progressive decline in circulating estrogen levels during menopausal transition can cause various symptoms. Genitourinary syndrome of menopause (GSM) is a common condition characterized by a series of menopausal symptoms. GSM is a new term that has replaced the formerly used vulvovaginal atrophy or atrophic vaginitis and is a more comprehensive term that includes vulvovaginal atrophy as well as lower urinary tract symptoms related to hypoestrogenic status [1].

Because the female genitalia and lower urinary tract have the same embryological origin, decreased circulating estrogen levels result in structural and functional changes in the epithelium and connective tissue of the urogenital area as well. Because of these changes, menopausal women experience not only vulvovaginal symptoms but also various urinary symptoms, such as urgency, dysuria and recurrent urinary tract infections. Moreover, these symptoms negatively affect sexual function in postmenopausal women. Although vasomotor symptoms typically improve over time, GSM has chronic and progressive characteristics that may have a profound impact on the quality of life of postmenopausal women.

Local hormonal treatments, especially low-dose vaginal estrogen tablets and creams, are considered the gold standard therapy for GSM and have demonstrated good efficacy in treating various symptoms and signs [2]. However, there are some obstacles in maintaining this effective treatment for menopausal women with GSM. Many patients complain of discomfort and inconvenience in inserting the tablets themselves, and the subsequent messiness after self-application results in poor compliance [3]. Owing to the chronic nature of GSM, long-term therapy is required. However, limited data are available regarding the long-term safety of these therapies [4].

Although vaginal estrogen therapy in postmenopausal women with a history of estrogen-dependent neoplasia is controversial, hormonal therapies improve GSM symptoms, sexual health and the quality of life of the patient; most recent guidelines recommend co-management of the patient with an oncologist [5]. After surgery, breast cancer survivors usually receive further adjuvant therapy, which can aggravate their vaginal symptoms [6].

Therefore, alternative treatment options must be considered. In recent years, intravaginal laser therapy has been suggested as a valuable non-hormonal therapeutic modality in the management of GSM.

Over the past 30 years, CO_2_ lasers have been developed and used for the treatment of a large number of skin and mucosal lesions [7,8]. The fractional CO_2_ laser has exhibited safety and remodeling of tissue properties in the skin of the face, neck and chest. Recently, studies on the efficacy and feasibility of the fractional CO_2_ laser in the treatment of GSM in menopausal women have been conducted, and the results have been quite promising. After laser treatment, the vaginal mucosa shows a much thicker epithelium with wide columns of glycogen-rich epithelial cells [9]. These changes may improve GSM symptoms. In addition, estrogen therapy is contraindicated for estrogen-dependent malignancies, such as breast cancer. Therefore, laser therapy could be an alternative treatment option for patients with breast cancer with GSM symptoms. 

This study aimed to investigate the safety and efficacy of fractional microablative CO_2_ laser treatment and its impact on sexual function in postmenopausal Korean women with GSM.

## 2. Materials and Methods

### 2.1. Study Population

This study was conducted after obtaining informed consent from the patients and as approved by the Institutional Review Board of Seoul St. Mary’s Hospital (KC20DESI1015). In this study, postmenopausal women with one or more symptoms related to GSM who were unresponsive to or unable to undergo traditional treatments, such as estrogen or local therapies, qualified for inclusion. Menopausal status can be spontaneous or iatrogenic. In this study, symptoms of GSM that patients may have suffered were defined based on the article by the North American Menopause Society and are as follows: dryness, burning and irritation; sexual symptoms of a lack of lubrication, discomfort or pain, and impaired function; and urinary symptoms of urgency, dysuria and recurrent urinary tract infection [1]. Patients with active genital infections, pelvic organ prolapse stage > II [10] or menopausal hormone therapy (systemic or local) history for up to 6 months before the study recruitment period were excluded from the study. Patients who used vaginal lubricants or any other local preparations were asked to discontinue the application of these treatments and were included in the study after 30 days.

### 2.2. Laser Treatment

Each patient received three laser applications with a fractional microablative CO_2_ laser (Smart Xide2; V2LR MonaLisa Touch System, DEKA, Florence, Italy) with an interval of 4 weeks between sessions, as follows: baseline (V1), 4-week follow-up (second laser application, V2), 8-week follow-up (third laser application, V3) and 12-week follow-up (V4). All laser applications were performed in an outpatient setting after local anesthesia gel application (Instillagel^®^, chlorhexidine gluconate solution; Falco-Pharma GmbH, Cologne, Germany). Because CO_2_ lasers have a strong affinity with water, we used only small amounts of anesthetic gel for coating vaginal introitus. During each session, the laser energy was set at 30 watts and transmitted through an intravaginal 360° probe with a dwelling time of 1000 μs, dot spacing of 1000 μm and a smart stack parameter of 1. This setting was based on previously published studies to ensure that excess energy was not transmitted [9,11].

### 2.3. Study Protocol

**Figure 1:**
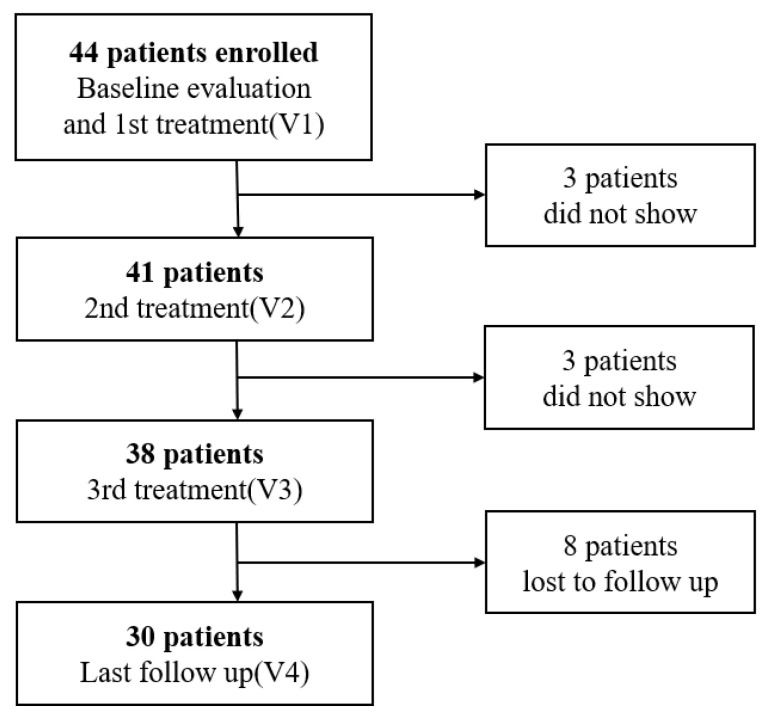
Study flow chart.

Before the first laser treatment at V1, the enrolled patients underwent gynecological examinations, including speculum insertion to measure vaginal elasticity and colposcopic inspection to check the integrity of the vaginal epithelium. Clinical data and questionnaires for objective indicators of treatment were collected at V1, V3 and V4 (Figure 1).

Several objective indicators were used to measure the severity of GSM. For analysis of the treatment outcomes, the intensity of GSM was recorded using a self-reported questionnaire based on the 10 cm Visual Analog Scale (VAS, 0–10) with 0 indicating the absence of symptoms, >0 and <4 indicating mild intensity of the symptoms, ≥4 and <8 indicating moderate intensity and ≥8 being severe. The questionnaire assessed vaginal dryness, burning sensations, itching, other discomforts, urinary difficulty, frequency and urgency. We used the Vaginal Health Index Score (VHIS), which is the sum of the following 5 components: elasticity, fluid volume, pH, epithelial integrity and moisture. Each component is scored from one (worst) to five (best). A score of ≤15 indicates the presence of vaginal atrophy [12].

Treatment satisfaction was evaluated 4 weeks after the last laser application (V4). Satisfaction was evaluated using a 5-point Likert scale (very satisfied, satisfied, uncertain, dissatisfied and very dissatisfied).

Any intraoperative complications (such as pain during probe insertion or burning and itching sensations) or postoperative complications (such as bleeding, pain, leukorrhea and discomfort) were recorded at any time.

### 2.4. Statistical Analysis

Continuous variables before and after treatment were analyzed using a paired *t*-test. Data are presented as mean ± standard deviation. Data were analyzed using SPSS software (version 21.0; SPSS Science, Chicago, IL, USA). A value of *p* < 0.05 was considered statistically significant. Statistical significance was set at *p* < 0.05.

## 3. Results

A total of 44 women were eligible for inclusion. Only 30 patients completed all the study protocols and the last follow-up observation a month later. Three women dropped out after the first laser therapy because they were already satisfied with the results and wanted to stop receiving laser therapy. Another three women dropped out after the second laser therapy for the same reason. Therefore, six women stopped laser therapy and did not have follow-up data. Eight women were lost to follow-up after completing the treatment course for the final observation. Thus, 30 patients completed the treatment with fractional CO_2_ lasers and all other procedures (Figure 1). 

The demographic characteristics of the study population are shown in Table 1. The mean age of the patients was 58 ± 5.5 years (range, 47–66 years) with a mean time of menopause at enrollment of 7.66 ± 4.2 years (range, 1–14 years). Eighty percent of the patients were diagnosed with breast cancer. Of the 30 patients included in this study, six experienced spontaneous menopause, and 24 experienced iatrogenic menopause secondary to oncologic treatment for breast cancer.

**Figure 2:**
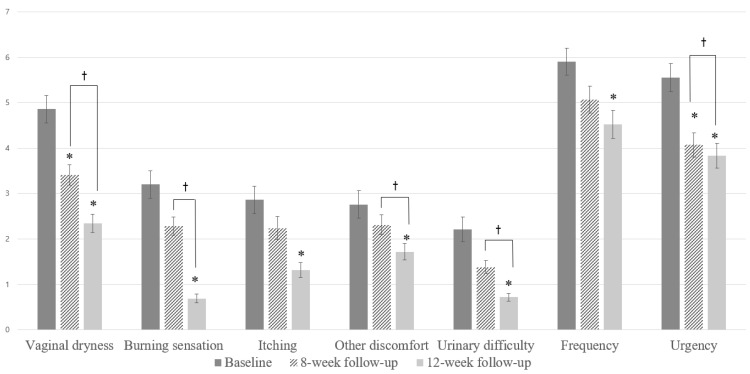
Symptoms of genitourinary syndrome of menopause (GSM). Assessment of the average change in GSM symptoms, including vaginal dryness, burning sensations, itching, other discomfort, urinary difficulty, frequency and urgency, was performed at baseline, 8-week follow-up (V3; after the second treatment) and 12-week follow-up (V4; at 1-month follow-up). Improvement was measured using a Visual Analog Scale score (0–10). * Statistically significant difference compared to baseline (*p* < 0.05); † Statistically significant difference between V3 and V4.

The intensity of GSM symptoms, including vaginal dryness, burning sensations, itching, other discomfort, urinary difficulty, frequency and urgency, was reported at V1, V3, and V4 (Figure 2). Vaginal dryness and urgency were significantly improved even after the second laser application, as compared to that at baseline (vaginal dryness: V1, 4.86 ± 3.07 vs. V3, 3.41 ± 2.24, *p* < 0.05; urgency: V1, 5.55 ± 3.15 vs. V3, 4.06 ± 2.77, *p* < 0.05). After completion of the third laser application, all GSM symptoms improved significantly at the 12-week follow-up compared with that at baseline (*p* < 0.05). Vaginal dryness, burning sensations, other discomfort, urinary difficulty and urgency were significantly improved after the third laser application compared to that after the second laser application (V3 vs. V4, *p* < 0.05). 

**Figure 3:**
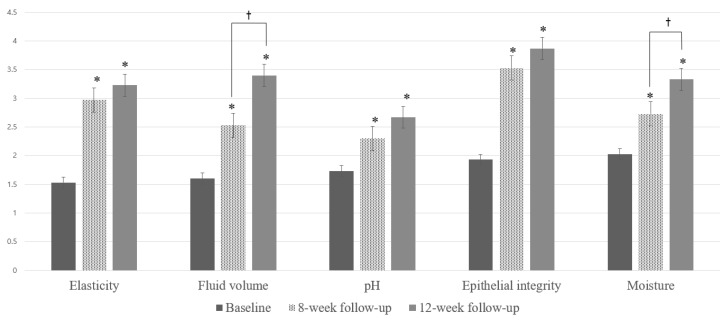
Vaginal health index score (VHIS). Assessment of the average change in VHIS at baseline, 8-week follow-up (V3; after the second treatment) and 12-week follow-up (V4; at 1-month follow-up). Improvement was measured using a value of 1 as the worst and 5 as the best. * Statistically significant difference compared to baseline (*p* < 0.001); † Statistically significant difference between V3 and V4 (fluid volume; *p* < 0.001, moisture; *p* < 0.05).

The VHIS also increased significantly after the completion of the treatment (total VHIS at baseline; 8.86 ± 3.2 vs. V3; 16.83 ± 3.15, *p* < 0.001). The mean changes in the VHIS are shown in Figure 3. The VHIS increased significantly in all components after treatment completion compared to the baseline (*p* < 0.001). Fluid volume and moisture were further improved significantly after the third laser treatment, compared with that after the second laser treatment (fluid volume: V3, 2.53 ± 0.62 vs. V4, 3.4 ± 0.93, *p* < 0.001; moisture: V3, 2.73 ± 0.78 vs. V4, 3.33 ± 0.66, *p* < 0.05).

Due to severe vaginal atrophy and very low cellularity in vaginal cytology, an evaluation of the Vaginal Maturation Index (VMI) was not possible. However, in some patients, an improvement in the inflammatory state was observed in vaginal cytology after laser treatment.

Four weeks after the last laser application, the average satisfaction score was 4.3 using a 5-point Likert scale. A total of 13 (43.3%) patients were very satisfied, 13 (43.3%) were satisfied, and 4 (13.4%) were uncertain about the laser procedure. 

After completion of the treatment, two patients complained of increasing vaginal discharge, and one patient reported mild pain lasting 1 to 2 days after treatment. However, none of the patients discontinued treatment because of these mild adverse events.

## 4. Discussion

The present study is one of the few trials conducted in Asia to demonstrate that fractional microablative CO_2_ lasers are an effective and safe treatment method for alleviating GSM symptoms. Similar to previous findings, this study proves that laser treatment is effective for almost all symptoms of GSM [11,13,14].

The data of this study ultimately provide evidence for the efficacy of lasers in improving the symptoms of vaginal dryness, burning and itching sensations, other vaginal discomforts, urinary urgency, urinary frequency and dysuria. Vaginal dryness and urgency improved after only two treatment sessions. In particular, the marked relief from vaginal dryness was statistically significant after each treatment session. The improvement in vaginal health was reflected not only by the subjective measurements of the 10 cm VAS score, but also by the significant improvement in objective indicators, such as the VHIS. All the VHIS components improved after the second and third laser sessions. Fluid volume and moisturization significantly improved between the second and third laser treatments. Results from the VHIS and VAS suggest that laser treatment is effective in relieving GSM and has enabled consistent improvements in vaginal dryness, as shown in previously published studies.

On 30 July 2018, the U.S. Food and Drug Administration (FDA) issued a warning regarding energy-based devices used to treat vaginal conditions and symptoms related to menopause, urinary incontinence or sexual function. However, many studies conducted prior to the publication of this warning did not show any suspicious results in terms of the effectiveness and safety of the treatments [15]. When this study began, the U.S. FDA issued a safety warning regarding vaginal laser treatment. Therefore, patients were notified of this issue prior to the procedure, and a survey was conducted on the possible adverse effects of each visit. If complications were suspected, patients could consult the researchers at any time. Thus, using these methods to check the possibility of all possible side effects and demonstrate the safety of laser treatment was the strength of this study. Throughout the process, three patients complained of discomfort during the laser treatment period, two of which were due to increased clear vaginal discharge. Only one patient reported mild vaginal pain within 1 to 2 days after treatment; however, the pain spontaneously disappeared and was limited to the first laser treatment session.

Although many studies have been conducted, they have rarely included Asian patients in their samples. According to previous studies, the severity of major postmenopausal symptoms associated with vaginal atrophy and sexual function after menopause varies greatly depending on the ethnic group [16,17]. In addition, there have been reports that the pelvic floor muscle is also affected by race [18,19]. Given the limited information available on Asian women in previous studies, this study is unique. Additionally, 80% of the patients enrolled in our trial were breast cancer survivors. We thus propose fractional CO_2_ laser treatment as an alternative treatment for patients with GSM and estrogen-dependent malignancies, such as breast cancer.

However, this study had several limitations. The sample size was relatively small, and the duration of the study was short, with limited long-term follow-up. Another limitation of this study was the absence of a control group that received either a placebo or other treatment modalities. However, this limitation is negligible given that the enrolled patients were unable to undergo estrogen therapy or did not respond to conventional treatments. Moreover, two gynecologists assessed the patients without knowing which session of the protocol each patient had completed. In addition, six women who dropped out before completing the third laser therapy session were already satisfied with the treatment results. Although the dropout rate was not very low, the reason for dropping out was not the side effects of the procedure. This is a promising aspect of laser therapy for the treatment of GSM.

Another limitation of this study is that the VMI value was not statistically significant. This is likely related to the chronically severe state of atrophic changes that patients experience for a long time without proper management. In some patients, an improvement in the inflammatory state can be detected via vaginal cytology. Takacs et al. considered that women with very low vaginal maturation values (VMV) respond differently to laser treatment compared to women with scores near or above the threshold levels [20]. However, in the group with no significant changes in VMV after laser treatment, vaginal dryness improved significantly from the baseline after the completion of laser treatment. Although VMI did not improve, laser treatment improved GSM symptoms.

The questionnaire included questions regarding the sexual function of those engaged in regular sexual relationships. However, with the cultural limitation of sexual relationships being regarded as a taboo, we could not use a validated questionnaire such as the Female Sexual Function Index or a simple VAS score.

Nevertheless, the results of this study are encouraging, given that women with GSM who are normally unresponsive to estrogen therapy experienced noticeable improvements in symptoms after laser treatment. This therapy was essentially painless and had no side effects. More importantly, notwithstanding the fact that the percentage of patients who dropped out appeared to be not very low, the cause of the patients’ dropout was not a result of the possible side effects correlated with the treatment. This study provides reasonable evidence for the use of laser therapy for managing GSM symptoms and encourages a long-term investigation in future.

## 5. Conclusions

The present study demonstrates that fractional microablative CO_2_ lasers offer an effective and safe treatment for GSM, with improvements in genitourinary symptoms. This finding is of great importance for women who are unable to use or are nonresponsive to vaginal estrogen therapy. Although the number of patients in this study was small and the follow-up period was not very long, this study establishes the basis for the validity and safety of the treatment in the current literature. Therefore, more extensive studies involving control groups are warranted.

## Figures and Tables

**Table 1:** Demographic characteristics of the study population (%).

Number (N)	30
Age (years)	58.3 ± 5.5
Years since last menstrual period	7.66 ± 4.2
Parity	1.83 ± 0.5
Previous history of breast cancer	24 (80%)
Adjuvant therapy with tamoxifen	10
Adjuvant therapy with aromatase inhibitors	9
Adjuvant therapy with herceptin	3
No adjuvant therapy	4
Sexually active women	13 (43.3%)

Data are presented as means ± SD or n.

## Data Availability

Data is unavailable due to privacy.
